# Pulmonary Hyalinizing Granuloma: A Rare Cause of a Benign Lung Mass

**DOI:** 10.3390/clinpract11010007

**Published:** 2021-01-29

**Authors:** Mandeep Singh Rahi, Kulothungan Gunasekaran, Kwesi Amoah, Farheen Chowdhury, Jeff Kwon

**Affiliations:** Division of Pulmonary Diseases and Critical Care Medicine, Yale-New Haven Health Bridgeport Hospital, 267 Grant Street, Bridgeport, CT 06258, USA; stankuloth@gmail.com (K.G.); kwesiamoah@gmail.com (K.A.); f.chowdhury@saba.edu (F.C.); Jeff.kwon@bpthosp.org (J.K.)

**Keywords:** lung mass, lung nodule, pulmonary hyalinizing granuloma, smoking

## Abstract

Pulmonary hyalinizing granuloma (PHG) is a rare, benign lung disease of unknown etiology. It usually manifests as solitary and sometimes as multiple pulmonary nodules. It may have irregular margins, cavitation, or calcifications mimicking metastasis or primary lung neoplasm. It should be considered in the differential diagnosis of pulmonary nodules or masses. In this report, we present an unusual case of incidental slow-growing lung mass in a patient with 30 pack-year smoking history, construction-based occupation. The pleural-based calcified nodule in the left upper lobe gradually increased in size over ten years without any hilar or mediastinal lymphadenopathy. For an accurate diagnosis, PET-scan and histopathological analysis through wedge resection by video-assisted thoracoscopic surgery (VATS) were done. The biopsy findings were consistent with pulmonary hyalinizing granuloma, a rare benign cause of lung mass with an excellent long-term prognosis.

## 1. Introduction

Pulmonary hyalinizing granuloma (PHG) is a benign and rare lung disease. It was first described by Benfield et al. in a 51-year-old female with retroperitoneal fibrosis and bilateral pulmonary granulomas [1]. It can present as single or multiple lung nodules. Most of the lesions are discovered incidentally as they are relatively asymptomatic. An accurate gauge of prevalence is hard to obtain. Peng et al. found one case of PHG in a cohort of 481 patients with lung disease [2]. Although the exact etiology is still unknown, it is hypothesized to be because of an exaggerated immune response to antigenic stimuli caused by infection or an autoimmune process [3,4]. The disease can mimic metastatic disease or primary lung malignancy, warranting appropriate diagnostic testing and management. Definitive diagnosis relies on histopathology showing central lamellar collagen sometimes arranged in whorls surrounded by giant cells and lymphocytes between the collagen bands [5]. Solitary nodule is usually managed effectively by complete resection. Surgery can be challenging and extensive in case of large lesions located close to critical structures. Whereas multiple lesions are difficult to manage surgically and may progress rapidly [6] We present a case of PHG describing characteristic calcification, histopathology, and growth over a period of ten years.

## 2. Case Presentation

**Figure 1:**
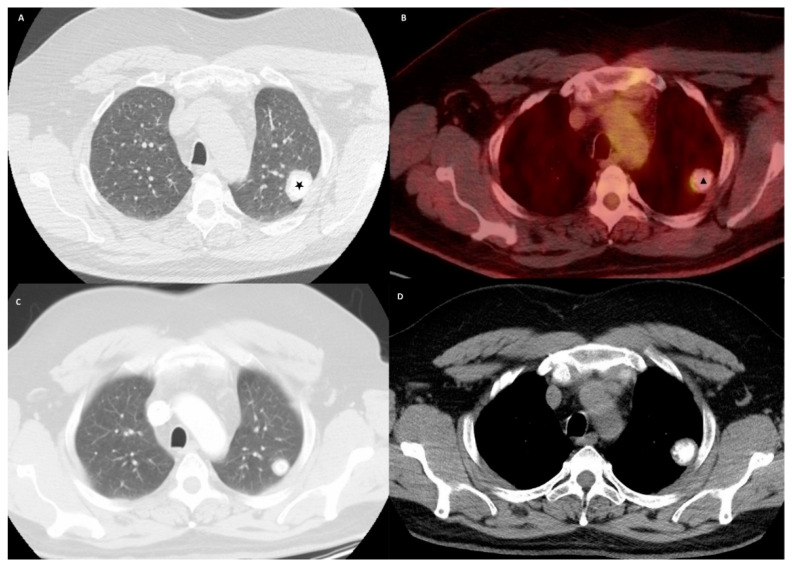
(**A**) is a computed tomography of chest in an axial plane showing a pleural based left upper lobe mass (denoted by a star), (**B**) is a positron emission tomography of chest in an axial plane showing mild avidity in the left upper lobe mass, (**C**) is computed tomography of chest in axial plane showing left upper lobe nodule, which is smaller in size ten years back, and (**D**) shows the left upper lobe nodule with central calcification.

**Figure 2:**
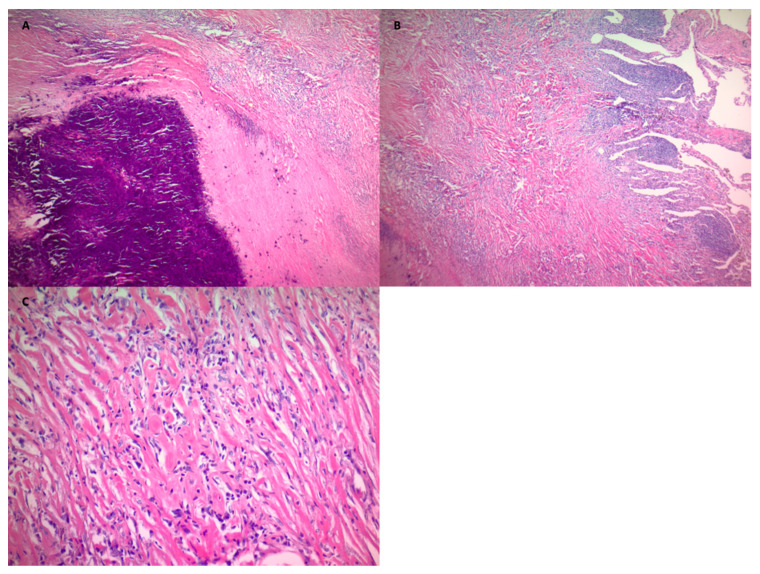
Is the histopathology of the resected mass from the left upper lobe showing dense network of hyalinized collagen and lymphocytic infiltrate (**A**,**B**). Collagen in lamellar pattern with whorls and surrounded by lymphocytic infiltrate (**C**) (Hematoxylin and Eosin staining, (**A**): ×40, (**B**): ×40, (**C**): ×400).

A 49-year-old male with a history of smoking presented to the emergency room with a one-week history of left shoulder pain, which started after mechanical trauma at his workplace. He endorsed burning micturition but denied fever, cough, shortness of breath, or chest pain. He was smoking one pack per day for the last 30 years. He was originally from Puerto Rico and moved to the mainland United States 20 years ago. He worked as a laborer in the construction business throughout his life. He had no known exposure to tuberculosis. On examination, he was afebrile with a blood pressure of 131/79 mm of Hg, heart rate of 89 beats per minute, respiratory rate of 18 breaths per minute, and oxygen saturation of 96% while breathing ambient air. Initial laboratory examination showed a white blood cell count of 17,800/μL, hemoglobin of 15.3 g/dL, and platelet count of 229 × 10_3_/μL. A plain radiograph of the left shoulder showed an incidental left upper lobe calcified nodule. Serum electrolytes and renal function tests were within normal limits. Urinalysis showed significant pyuria with moderate bacteria. Urine culture grew Escherichia coli sensitive to beta-lactam antibiotics, and he was treated with antibiotics. For evaluation of the lung nodule, a Computed Tomography (CT) of the chest was obtained, which showed a 3.2 × 2.2 cm pleural-based mass with central calcification in the left upper lobe with no mediastinal or hilar lymphadenopathy (Figure 1A,D). A careful review of the chart showed that he had the same nodule ten years back, but it was only 1.7 cm (Figure 1C). The patient was discharged and was followed in the out-patient clinic. A Positron Emission Tomography (PET) scan was performed, and the pulmonary nodule showed mild avidity with a standardized uptake value (SUV) max of 3.1 (Figure 1B). The patient was discussed in a multispecialty tumor board conference, and the consensus was to favor resection due to the size of the mass, growth over the last ten years, relatively young age, and possibility of the increased complexity of resection with further growth in the tumor. His pulmonary function tests showed moderate obstruction with a normal gas transfer. The patient underwent wedge resection by Video-Assisted Thoracoscopic Surgery (VATS). Pathology showed a focally calcified nodule with dense lamellar collagen consistent with hyalinized granuloma (Figure 2). No amyloid fibers were seen, and stains were negative for acid-fast and fungal organisms. The patient had an unremarkable postoperative course and was discharged after 48 h. There were no complications reported on the follow-up visit and no recurrence on surveillance imaging one year post-operatively.

## 3. Discussion

Pulmonary Hyalinizing Granuloma is a benign fibro-sclerosing pulmonary nodule consisting of concentric hyaline lamellae that could present as multiple, bilateral, or mildly symptomatic lesions, showing characteristics of amyloid or atypical birefringence patterns [3,4]. Although the exact etiology is still unknown, it is hypothesized to be from an aberrant immune response to various infectious antigens (fungal and mycobacterial), systemic fibrosis, and an autoimmune process like sarcoidosis, grave’s disease, cutaneous vasculitis, and multiple sclerosis [3,7,8] No specific gender or race has been shown to be at risk. There is some propensity to develop it between the ages of 15–77 years, with a mean age of presentation at 44 years [3,9]. Clinical manifestations with this disease can vary from non-specific symptoms such as cough, fever, shortness of breath, or chest pain to the incidental finding of a nodule or mass on a chest radiograph in approximately 25% of patients [10].

The differential diagnoses include autoimmune diseases like Wegener’s granulomatosis, rheumatoid nodules, idiopathic thrombocytopenic purpura, sarcoidosis, TB, amyloidosis, fungal infections, and inflammatory myofibroblastic tumors [11]. Autoimmune conditions are linked to PHG as there are reports about patients with raised rheumatoid factor levels, anti-nuclear and anti-globulin antibodies [12]. It is also considered to be related to other fibro-sclerosing conditions such as retroperitoneal fibrosis, sclerosing mediastinitis, and sclerosing cholangitis [10]. Some associations of PHG with lymphoproliferative diseases like lymphoma and Castleman’s disease has also been demonstrated [13]. More importantly, it can mimic a primary lung cancer or metastatic disease warranting due investigation and management. Unlu et al. described a patient with PHG whose radiological images showed the appearance of metastatic lung cancer [14]. In another study by Lhote et al., PHG was found to be associated with tumors in six cases, including carcinomas of the breast, lung, thyroid, Paget’s disease, meningioma, anaplastic astrocytoma, and basocellular carcinoma [8]. Similarly, Ren et al. reported an association between PHG and lymphoma [15].

Imaging is usually the first step in characterizing the lesion. Typically, plain radiographs and CT of the chest show PHG as solitary or multiple randomly distributed pulmonary nodule(s) or mass that is distributed unilaterally or bilaterally without preference for any particular parenchymal location [16]. Adenopathy is usually not associated with the disease unless the patient has an underlying active infectious, mycobacterial, or granulomatous process like histoplasmosis. Follow-up imaging can sometimes show slow growth and coalescing tendencies overtime [9]. PET scans could help to differentiate from malignant tumors. But studies have shown that up to 60% of PHG lesions show increased metabolic activity using fluorodeoxyglucose (FDG)–PET scan and can be false positive. An accurate diagnosis of PHG relies mostly on histopathological examination [10,16].

The PHG nodules are classically described as well-defined and homogenous, ranging in size from several millimeters to 15 cm in diameter, the average being 2 cm. They can calcify and are rarely reported to cavitate [3]. Calcification in pulmonary nodules is generally considered to point towards a benign process, as long as follow-up with computed tomography shows no growth for two years. However, Khan et al. described how calcification in a pulmonary nodule as a criterion to establish it as being of a benign nature could be fallacious and sometimes misleading. Differentials to be considered for a calcified lesion other than granulomas like PHG include hamartomas, carcinoid, chondrosarcomas, osteosarcomas, lung metastases, or primary bronchogenic carcinoma, among others [17].

The next step is usually obtaining a tissue diagnosis. Given the peripheral location, traditional bronchoscopy with trans-bronchial biopsies does not lead to an accurate diagnosis [8]. A CT-guided percutaneous biopsy or surgical resection is recommended to confirm the diagnosis [6,18]. On histopathological examination, lesions are characterized by a dense network of hyalinized collagen in the center of perivascular lymphoplasmacytic infiltrate, wherein the collagen is deposited in ropy, whorled bundles separated by clear spaces and surrounded by giant cells [19]. Congo red stain with polarized light and crystal violet stain is usually negative in PHG but will be positive in cases of Amyloidosis. In PHG, the hyaline lamellae are more compact and amorphous, which is very different from the fibrillar lesions of Amyloidosis [7,16].

PHG has growth potential as well, but the doubling-time is variable [4]. Surgical resection is diagnostic and usually curative. Solitary or peripheral lesions are generally amenable to VATS, which is a less invasive procedure. For patients with multiple pulmonary nodules, surgical resection is usually not feasible. Although unclear in their therapeutic benefit, corticosteroids have been used in the past in patients with multiple nodules due to PHG [3]. Malignant potential is unknown, and it may still recur following surgical removal [3]. In one review, 73 patients with PHG were followed, out of whom 14 were treated with surgical resection, and 5 had a recurrence. 46 patients were treated with corticosteroids, and a majority had stable disease. Long term prognosis of PHG is usually excellent with continued surveillance.

## 4. Conclusions

PHG is a rare diagnosis; therefore, patients with risk factors for malignancy, based on their smoking history, age or occupation, and similar radiologic presentations to cancer, need to be evaluated appropriately. It is important to note that the absence of FDG uptake on PET scan should not sway away from seeking a histopathological diagnosis in high-risk patients. Other patients may be followed with close surveillance. PHG should be included in the differential diagnosis of patients with pulmonary nodules. Even though benign, most of the patients would need excision biopsy to rule out other malignant conditions.
